# Rectal Microbiome Composition Correlates with Humoral Immunity to HIV-1 in Vaccinated Rhesus Macaques

**DOI:** 10.1128/mSphere.00824-19

**Published:** 2019-12-11

**Authors:** Sonny R. Elizaldi, Anil Verma, Korey A. Walter, Matthew Rolston, Ashok R. Dinasarapu, Blythe P. Durbin-Johnson, Matthew Settles, Pamela A. Kozlowski, Reben Raeman, Smita S. Iyer

**Affiliations:** a Graduate Group in Immunology, University of California, Davis, California, USA; b The Center for Immunology and Infectious Diseases, University of California, Davis, California, USA; c Department of Microbiology, Immunology, and Parasitology, Louisiana State University Health Sciences Center, New Orleans, Louisiana, USA; d Host Microbe Systems Biology Core, University of California, Davis, California, USA; e Department of Human Genetics, Emory University, Atlanta, Georgia, USA; f Division of Biostatistics, School of Medicine, University of California, Davis, California, USA; g UC Davis Genome Center, Davis, California, USA; h Department of Pathology, University of Pittsburgh, Pittsburgh, Pennsylvania, USA; i Pittsburgh Liver Research Center, University of Pittsburgh, Pittsburgh, Pennsylvania, USA; j California National Primate Research Center, School of Veterinary Medicine, Davis, California, USA; k Department of Pathology, Microbiology, and Immunology, School of Veterinary Medicine, University of California, Davis, California, USA; National Institute of Allergy and Infectious Diseases

**Keywords:** vaccine, DNA, HIV-1, antibody response, microbiome

## Abstract

The microbiome is an integral and dynamic component of the host and is emerging as a critical determinant of immune responses; however, its influence on vaccine immunogenicity is largely not well understood. Here, we examined the pivotal relationship between the mucosal microbiome and vaccine-induced immune responses by assessing longitudinal changes in vaginal and rectal microbiome profiles after intradermal immunization with a human immunodeficiency virus type 1 (HIV-1) DNA vaccine in adult rhesus macaques that received two prior DNA primes. We report that both vaginal and rectal microbiomes were dominated by *Firmicutes* but were composed of distinct genera, denoting microbiome specialization across mucosal tissues. Following immunization, the vaginal microbiome was resilient, except for a transient decrease in *Streptococcus*. In contrast, the rectal microbiome was far more responsive to vaccination, exhibiting an increase in the ratio of *Firmicutes* to *Bacteroidetes*. Within *Bacteroidetes*, multiple genera were significantly decreased, including *Prevotella, Alloprevotella, Bacteroides, Acetobacteroides, Falsiporphyromonas*, and *Anaerocella.* Decreased abundance of *Prevotella* correlated with induction of gut-homing α_4_β_7_^+^ effector CD4 T cells. *Prevotella* abundance also negatively correlated with rectal HIV-1 specific IgG levels. While rectal *Lactobacillus* was unaltered following DNA vaccination, baseline *Lactobacillus* abundance showed strong associations with higher rectal HIV-1 gp140 IgA induced following a protein boost. Similarly, the abundance of *Clostridium* in cluster IV was associated with higher rectal HIV-1 gp140 IgG responses. Collectively, these data reveal that the temporal stability of bacterial communities following DNA immunization is site dependent and highlight the importance of host-microbiome interactions in shaping HIV-1 vaccine responses. Our findings have significant implications for microbial manipulation as a strategy to enhance HIV vaccine-induced mucosal immunity.

**IMPORTANCE** There is considerable effort directed toward evaluating HIV-1 vaccine platforms to select the most promising candidates for enhancing mucosal HIV-1 antibody. The most successful thus far, the RV144 trial provided partial protection due to waning HIV-1 antibody titers. In order to develop an effective HIV vaccine, it may therefore be important to understand how biological factors, such as the microbiome, modulate host immune responses. Furthermore, as intestinal microbiota antigens may generate antibodies cross-reactive to the HIV-1 envelope glycoprotein, understanding the relationship between gut microbiota composition and HIV-1 envelope antibody responses after vaccination is important. Here, we demonstrate for the first time in rhesus macaques that the rectal microbiome composition can influence HIV-1 vaccine immunogenicity, and we report temporal changes in the mucosal microbiome profile following HIV-1 vaccination. Our results could inform findings from the HIV Vaccine Trials Network (HVTN) vaccine studies and contribute to an understanding of how the microbiome influences HIV-1 antibody responses.

## Abstract (precis)

There is considerable effort directed toward evaluating HIV-1 vaccine platforms to select the most promising candidates for enhancing mucosal HIV-1 antibody. The most successful thus far, the RV144 trial provided partial protection due to waning HIV-1 antibody titers. In order to develop an effective HIV vaccine, it may therefore be important to understand how biological factors, such as the microbiome, modulate host immune responses. Furthermore, as intestinal microbiota antigens may generate antibodies cross-reactive to the HIV-1 envelope glycoprotein, understanding the relationship between gut microbiota composition and HIV-1 envelope antibody responses after vaccination is important. Here, we demonstrate for the first time in rhesus macaques that the rectal microbiome composition can influence HIV-1 vaccine immunogenicity, and we report temporal changes in the mucosal microbiome profile following HIV-1 vaccination. Our results could inform findings from the HIV Vaccine Trials Network (HVTN) vaccine studies and contribute to an understanding of how the microbiome influences HIV-1 antibody responses.

## INTRODUCTION

Generation of robust and durable cellular and humoral immune responses constitute the fundamental basis of vaccine-mediated protection from infectious disease (1). Immune responses elicited by vaccination are, however, heterogenous which results in variable vaccine efficacy at the population level, a phenomenon attributed to the complex interplay between host intrinsic factors such as age, sex, genetics, diet, and lifestyle, and their interactions with the immune system (2). Discerning the relationship between host intrinsic factors and vaccine-induced immune responses will facilitate a better understanding of the determinants of vaccine efficacy and inform vaccine efforts against challenging infectious diseases, such as human immunodeficiency virus (HIV) (3).

Microbiota, a polymicrobial community dominated by bacteria and encompassing archaea, fungi, protozoa, and viruses, are emerging as critical host determinants of immunity (4, 5). Recent studies demonstrate that the microbial community structure within the gut not only regulates immune cell development but also influences immune responses after antigen challenge (6, 7). The importance of the microbiome in shaping the immune landscape is exemplified in germfree mice, which show impaired cellular level deficits in the development of gut-associated lymphoid tissues (8, 9). Compared to animals housed under specific-pathogen-free conditions, the absence of a stable microbiome community in germfree mice results in fewer germinal centers within mesenteric lymph nodes and reduced frequencies of CD4 T regulatory (Treg) cells (8); the latter is attributed to the absence of *Clostridium* species that drive Treg differentiation (10). Dysregulated CD4 T helper T_h_17 cell responses in the intestinal lamina propria are also observed in germfree mice, due to the absence of segmented filamentous bacteria which mediate T_h_17 polarization of CD4 T cells (11, 12). In addition to defects in lymphocyte development, germfree mice have impaired adaptive immune responses to infections. This defect is largely due to decreased trafficking of antigen-experienced CD4 T cells to sites of infection and the failure to mount strong systemic antigen-specific responses (13–15). In line with the profound immune deficiencies in germfree mice, antibiotic treatment, which disrupts native microbial community structure, is shown to undermine immune responses. Gut microbial dysbiosis of infant mice, resulting from antibiotic treatment, impairs antigen-specific serum IgG titers in response to live and adjuvanted vaccines (16).

In line with mouse studies, which underscore the nexus between the microbiome and host immunity, there is compelling evidence in both humans and nonhuman primates that the microbiome directs humoral immune responses to vaccination (17–19). Studies in humans show that an abundance of probiotic bacterial strains can augment cellular and humoral responses to oral and parenterally delivered vaccines (16, 18). Rhesus macaques supplemented with probiotics containing *Bifidobacterium* and *Lactobacillus* spp. displayed increased frequencies of colonic IgA-positive (IgA^+^) B cells and lymph node CD4 T_FH_ cells, revealing a potential mechanistic basis for microbiota-dependent enhancement of humoral immunity (20). These studies demonstrate the plasticity of the gut microbiome and the feasibility of targeting the microbiome to enhance immune responses. Therefore, understanding the positive and negative interactions of bacterial species with host immune function is necessary, especially against challenging pathogens such as HIV. However, we know relatively little about how nonreplicating immunogens such as DNA vaccines, routinely used to prime immune responses to HIV type 1 (HIV-1), modulate the mucosal microbiome.

The objectives of the present study were to determine the influence of DNA vaccine-induced immunomodulation on the rectal microbiome composition and to assess whether the rectal microbiome composition is associated with DNA vaccine-primed HIV-1 antibody responses. To this end, we evaluated rectal microbiome profile at weeks 0, 1, and 4 after third DNA immunization (DNA3) priming immunization in adult female rhesus macaques. Our data show that the frequencies of gut-homing CD4 T cells following vaccination are negatively associated with the abundance of *Prevotella*, and strikingly, *Prevotella* abundance is negatively associated with rectal HIV-1 IgG. These dynamic changes in *Prevotella* were not observed following live measles booster immunization, indicating specificity of microbial malleability to DNA vaccine-induced immunomodulation. We found that relative abundance of *Lactobacillus* and *Clostridium* IV (*Clostridium* in cluster IV) spp. positively correlated with rectal HIV-1-specific IgA and IgG responses, respectively. Taken together, our findings reveal that (i) DNA vaccine-induced immune responses have the capacity to modulate rectal microbial profiles in rhesus macaques and (ii) specific microbiota associate with HIV-1 gp140 antibody concentrations. These findings provide a rationale to investigate how distinct microbial taxa may be manipulated to improve HIV-1 vaccine immunogenicity, particularly to enhance antibody durability.

## RESULTS

### Study design for microbiome profiling.

**FIG 1:**
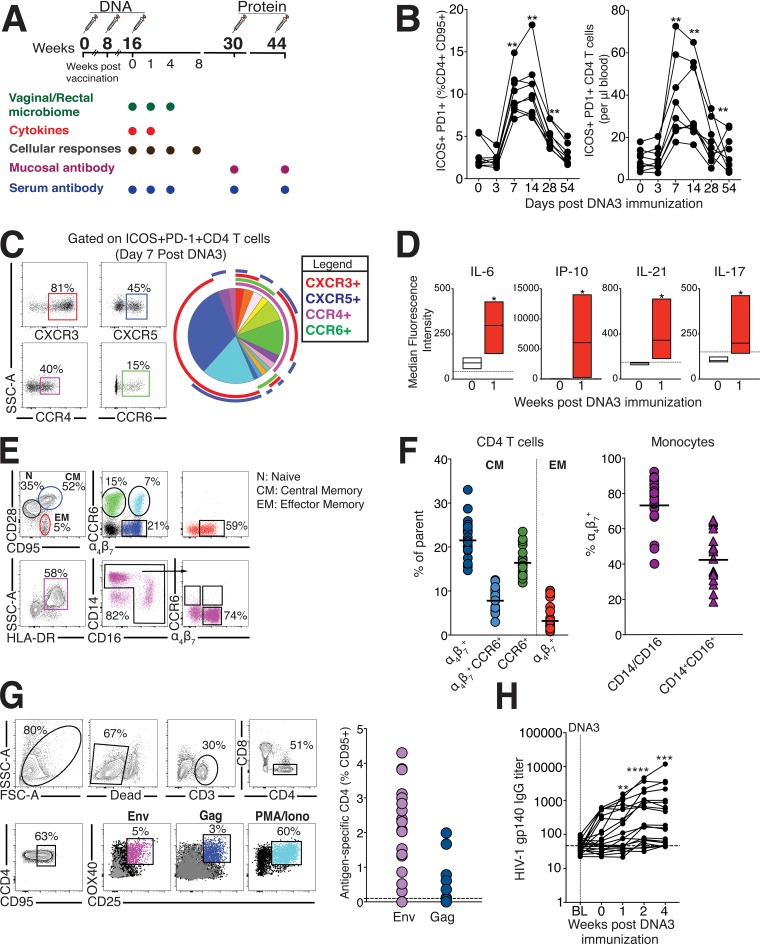
Study design and immune responses after HIV-1 DNA/protein immunization. (A) Study overview. Adult female rhesus macaques (*n* = 20) were immunized with HIV-1 DNA plasmid (three times) followed by immunization with HIV-1 gp140 protein (two times). Samples for microbiome profiling using 16S rRNA gene sequencing were collected at weeks 0, 1, and 4 of DNA3 immunization. Cellular and antibody responses were assessed at the indicated time points. (B) Transient accumulation of ICOS^+^ PD-1^+^ CD4 T cells expressed as relative frequencies (left) and absolute counts (right) in blood. (C) Gating strategy to evaluate chemokine receptor expression on activated CD4 T cells in blood on day 7 after DNA3 immunization. Boolean-gated frequencies within FlowJo were exported into SPICE to generate a pie chart that shows that the majority of activated CD4 T cells were CXCR3^+^, CXCR3^+^ CXCR5^+^, and CCR4^+^ CXCR3^+^. (D) Induction of IL-6, IP-10, IL-21, and IL-17 in serum 7 days after DNA3 immunization. (E and F) Expression of CCR6 and α_4_β_7_ on central memory (CM) and effector memory (EM) CD4 T cells and DR^+^ CD14/CD16 monocytes (circles) and CD14^+^ CD16^+^ proinflammatory monocytes (triangles). (G) Gating strategy to identify OX40^+^ CD25^+^ Env and Gag-specific CD4 T cells within peripheral blood mononuclear cells (PBMCs) after stimulation. Scatter plots show frequencies of antigen-specific CD4 T cells after background subtraction. FSC, forward scatter; PMA, phorbol myristate acetate; Iono, ionomycin. (H) Kinetics of HIV-1 specific IgG titers in sera following DNA3. BL, baseline. Statistical significance over time was tested using the Mann-Whitney U test; *, *P* ≤ 0.05; **, *P* ≤ 0.01; ***, *P* ≤ 0.001; ****, *P* ≤ 0.0001.

We longitudinally collected matched vaginal and rectal secretions from 20 adult female macaques before and after immunization with a DNA plasmid expressing HIV-1 Env and simian immunodeficiency virus SIV239 Gag, given intradermally with electroporation. We evaluated microbiome composition at weeks 0, 1, and 4 after DNA3 priming immunization to address two questions. (i) Is the mucosal microbiome altered after DNA immunization? (ii) Does the mucosal microbiome profile at week 0 and week 1 of DNA3 prime correlate with mucosal antibody responses at the effector (2 weeks) and memory time points (8 and 16 weeks) following the protein boost? HIV-1 specific CD4 T cell and antibody responses are detectably primed at DNA3, enabling us to not only determine stability of the microbiota to perturbations arising from immune responses but also determine associations between microbiota and humoral immunity. We used a combination of cellular, cytokine, and antibody measurements to evaluate immune responses at the indicated time points (Fig. 1A).

### Immune responses following DNA prime immunization.

Given that DNA immunizations prime stronger CD4 T cell responses relative to CD8 T cells, we sought to identify activated CD4 T cells by assessing expression of inducible costimulator (ICOS) and programmed death 1 (PD-1), cell-surface markers induced upon T cell receptor (TCR) stimulation (21). We observed expansion of ICOS^+^ PD-1^+^ CD4 T cells 7 days after vaccination (*P* < 0.01) with elevated frequencies at day 14 (*P* < 0.01; Fig. 1B). Both the relative frequency and absolute counts of ICOS^+^ PD-1^+^ CD4 T cells in blood were transiently increased at day 7, 14, and 28 time points, corresponding to the effector phase of the immune response.

To gain insights into the differentiation profile and trafficking potential of DNA vaccine-induced CD4 T cell effectors, we phenotyped ICOS^+^ PD-1^+^ CD4 T cells at day 7 for expression of chemokine receptors: CXCR3 [chemokine (C-X-C motif) receptor 3] (T_h_1), CXCR5 (T_fh_), CCR4 [chemokine (C-C motif) receptor 4] (T_h_2), and CCR6 (T_h_17) (Fig. 1C). The majority of effectors (32.4%) showed exclusive expression of CXCR3, which directs migration to inflammatory sites and to the vaginal mucosa (22). The next abundant subset (18%) coexpressed CXCR3 and CXCR5, suggesting induction of T_h_1-skewed T_FH_ cells (23). We also observed coinduction of T_h_2 and T_h_17 cells, suggesting that polyfunctional CD4 responses were elicited following DNA vaccination. The robust induction of T_h_1, T_fh_, T_h_17 cytokines interleukin-6 (IL-6), interferon protein 10 (IP-10), IL-21, and IL-17 was consistent with the observed T_h_1/T_fh_/T_h_17 differentiation profile of CD4 T cells and denoted the induction of an inflammatory response following DNA3 priming immunization (Fig. 1D). To more systematically assess the gut-homing potential of CD4 T cells during the effector phase, we determined coexpression of integrin α_4_β_7_ and CCR6, receptors that direct migration of cells to gut-associated lymphoid tissue and the mucosal epithelium, respectively (24). CD28^+^ CD95^+^ central memory CD4 T cells displayed robust expression of α_4_β_7_, with a third of α_4_β_7_^+^ cells coexpressing CCR6. In contrast, CD28-negative (CD28^−^) CD95^+^ effector memory (EM) cells exclusively expressed α_4_β_7_ with relatively little CCR6 expression, as did monocytes (Fig. 1E and F).

Evaluation of antigen-specific responses to vaccine immunogens, Env and Gag, at day 7 after DNA3 immunization revealed induction of Env-specific CD4 T cells in a majority of animals, while responses to Gag were sporadically observed (Fig. 1G). Consistent with availability of antigen-specific CD4 T cell help for humoral immunity, we observed induction of anti-gp140 HIV-1 envelope (Env) antibody responses following DNA3 immunization (Fig. 1H). We also detected robust HIV-1 gp140 Env-specific responses in sera and rectal secretions following protein boost (see Fig. S1 in the supplemental material). Together, these data demonstrate that DNA immunization establishes an inflammatory response, elicits CD4 effector T cells with the potential to home to gut/vaginal mucosa, and primes antigen-specific cellular and humoral responses, which are robustly boosted following protein immunization.

10.1128/mSphere.00824-19.2 FIG S1 Kinetics of HIV-1 gp140 IgG in sera and HIV-1 gp140 IgA/IgG specific activity in rectal secretions (*n* = 20 animals; median shown). Statistical significance across time was tested using the Mann-Whitney U test; **, *P* ≤ 0.01; ***, *P* ≤ 0.001; ****, *P* ≤ 0.0001. Download FIG S1, TIF file, 0.9 MB. Copyright © 2019 Elizaldi et al. 2019 Elizaldi et al. This content is distributed under the terms of the Creative Commons Attribution 4.0 International license.

### Rectal and vaginal mucosal niches have similar representation of bacterial phyla but are composed of distinct bacterial genera.

Prior to investigating whether immune responses following DNA immunization were associated with changes in the mucosal microbiome profile, we assessed compositional similarities and differences between bacteria present in the vaginal and rectal compartments at baseline (week 0 of DNA3 immunization). The collection of paired cervicovaginal lavage (CVL) samples and rectal sponges from our cohort of 20 animals along with contemporaneous processing and sequencing of extracted DNA provided a powerful opportunity for robust comparative assessments across these distinct mucosal compartments. Furthermore, CVL samples and rectal sponges were, on average, sequenced at the same depth and produced similar numbers of high-quality reads, indicating effective recovery and amplification of bacterial DNA by both sampling techniques.

We obtained 3,832,685 high-quality reads from CVL samples and 4,573,222 bacterial reads from rectal sponges. Phyla with a mean prevalence of <5% and ambiguous phyla (phyla with no phylum-level taxonomy or phylum listed as “uncharacterized”) were removed. Data were aggregated at the genus level, and all taxa without genus-level taxonomic assignments were removed. The final CVL data set had 268 operational taxonomic units (OTUs) and a read range of 20,343 to 141,526 reads. The final rectal microbiome data set had 324 OTUs and a read range of 17,277 to 116,378. High-quality reads were classified using Silva 132 as the reference database.

**FIG 2:**
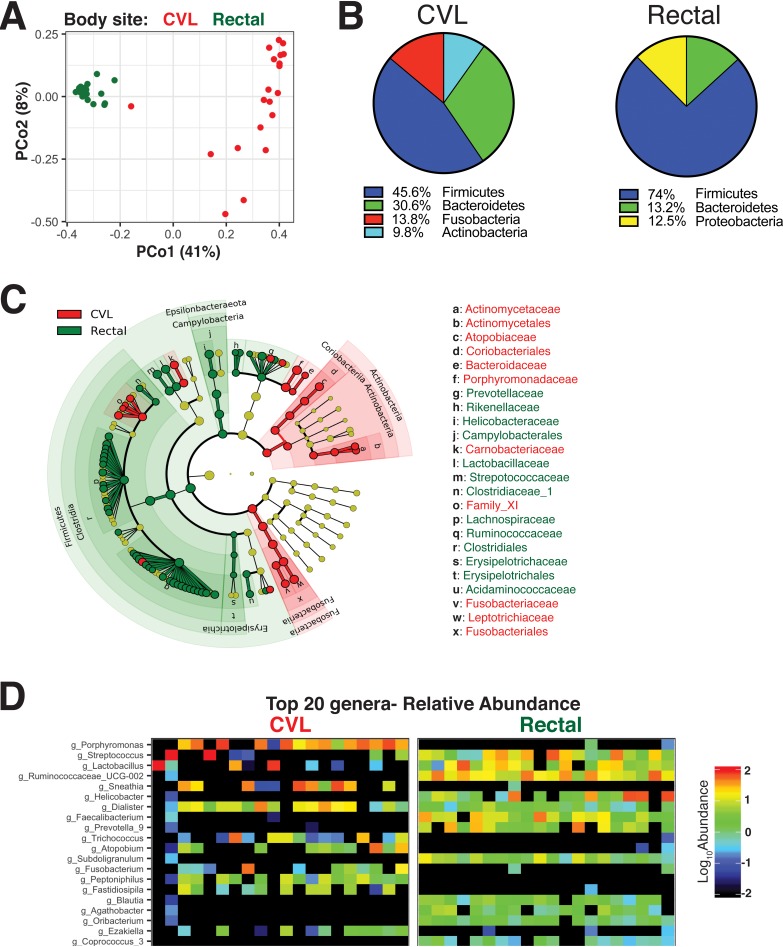
Diversity of microbial community composition across rectal and vaginal compartments. (A) 16S rRNA sequence data from paired cervicovaginal lavage (CVL) samples and rectal sponges showed unique clustering evident from principal-coordinate analysis (PCoA) plot based on unweighted UniFrac distances between bacterial communities across each mucosal site at week 0. (B) Evaluation of communities at the phylum level showed dominance of *Firmicutes* in both compartments. (C) Cladogram representing taxa significantly different between compartments by LEfSe analysis. (D) Heat map of the top 20 most abundant genera in CVL and rectal compartments show the presence of unique phylotypes in each compartment not found in the other. Each column represents data from a single animal.

Shannon alpha-diversity was estimated using a linear mixed-effects model with a random intercept for animal. The model revealed no significant changes in alpha-diversity over time within the vaginal and rectal compartments. As illustrated in the principal-coordinate analysis (PCoA) plot, the vaginal compartment demonstrated, on average, a higher variability in microbiome composition relative to the rectal microbiome (Fig. 2A).

At the phylum level, rectal bacterial communities were composed of *Firmicutes*, *Bacteroidetes*, and *Proteobacteria* with *Firmicutes* representing the most predominant phylum with a median relative abundance of 74% (Fig. 2B), reflecting the characteristic dominance of *Firmicutes* and *Bacteroidetes* in the human and mouse gut (25, 26). *Firmicutes* were also enriched within the vaginal compartment accounting for 45.6% (median) of bacteria sequenced, followed by *Bacteroidetes* (30.6%), *Fusobacteria* (13.8%), and *Actinobacteria* (9.8%). While *Fusobacteria* and *Actinobacteria* were minor constituents within the rectal compartment, at a relative abundance of <1%, the vaginal compartment was entirely lacking in organisms of the *Proteobacteria* phylum.

Because *Firmicutes* are the most diverse of the bacterial phyla, we next asked whether rectal and vaginal (CVL) microbiota showed compositional differences at lower taxonomic levels. As demonstrated by the linear discriminant analysis effect size (LEfSe) plot, microbiome in vaginal (CVL) and rectal compartments demonstrated characteristic phylotype abundances (Fig. 2C). Gram-positive commensal *Clostridia* and its family *Ruminococcaceae* were among the most predominant in the rectum, while Gram-negative *Fusobacteria and Bacteroidia* and its family *Porphyromonadaceae and* Gram-positive *Actinobacteria* were among the most abundant within the vaginal compartment. The representation of Gram-positive obligate anaerobes of the family *Ruminococcaceae* in the rectum is expected based on the high carbohydrate content (56% calories from carbohydrate) in the captive rhesus diet (27). Within Gram-positive *Bacilli*, members of the family *Streptococcaceae* and *Lactobacillaceae* dominated in both the rectal and vaginal (CVL) compartments with *Carnobacteriaceae* being exclusively represented in CVL samples at frequencies of greater than 1%.

Evaluation of microbial composition at the genus level revealed that the gut and vaginal microbiota were comprised of distinct phylotypes (Fig. 2D and Fig. S2). In the CVL samples, at the genus level, *Porphyromonas* predominated, followed by the genus *Dialister* of the family *Veillonellaceae*; *Lactobacillus*, and *Streptococcus* (phylum *Firmicutes* ), and *Sneathia* of the phylum *Fusobacteria*. *Lactobacillus* was observed at a relative abundance of above 1% in only 3/20 animals sampled, and notably, animals with a predominance of *Lactobacillus* showed lower absolute abundance of *Porphyromonas* (*r* = −0.5, *P* < 0.01), which is consistent with the observation that low *Lactobacillus* abundance facilitates growth of *Porphyromonas* and *Sneathia* by altering substrate availability (28). Indeed, a classical feature of bacterial vaginosis in humans is a shift from dominant *Lactobacillus* species to a more diverse population which includes pathogenic bacteria such as *Prevotella* spp. (29). In the gut, *Streptococcus* and *Helicobacter* genera dominated, followed by *Prevotella*, *Lactobacillus*, and *Faecalibacterium*. The absence of *Helicobacter* within the CVL samples and *Atopobium* (phylum *Actinobacteria*) in the gut denoted the presence of unique bacterial communities in each body site. Together, these data demonstrate that the vaginal and gut microbiota have phylum-level similarities with an abundance of *Firmicutes* but display distinct genus-level repertoires (Fig. 2D and Fig. S3A). We next asked whether cohousing or sibship associated with rectal and vaginal microbiome profiles. We found that samples from the same housing unit or from related animals did not appear more similar to each other than would be expected by random chance (Fig. S3B and C). We also asked whether vaginal microbiome clustered by time since menses and found no significant associations between the two variables (Fig. S4). Collectively, our data show that the rectal and vaginal compartments are distinct and composed of vastly diverse genera.

10.1128/mSphere.00824-19.3 FIG S2 Heat map shows top 50 differentially abundant genera in CVL versus rectal compartments. Each column represents a single animal. Download FIG S2, TIF file, 4.6 MB. Copyright © 2019 Elizaldi et al. 2019 Elizaldi et al. This content is distributed under the terms of the Creative Commons Attribution 4.0 International license.

10.1128/mSphere.00824-19.4 FIG S3 (A) Contingency plots showing the top 10 differentially abundant taxa in CVL and rectal compartments. (B and C) Hierarchical clustering dendrograms using Bray distances of rectal microbiome (B) and vaginal microbiome (C) shows no significant association with cohousing or sibship. Dendrograms are colored by housing unit and sibship. Download FIG S3, TIF file, 2.2 MB. Copyright © 2019 Elizaldi et al. 2019 Elizaldi et al. This content is distributed under the terms of the Creative Commons Attribution 4.0 International license.

### Temporal dynamics in vaginal microbiome composition following HIV-1 DNA immunization.

**FIG 3:**
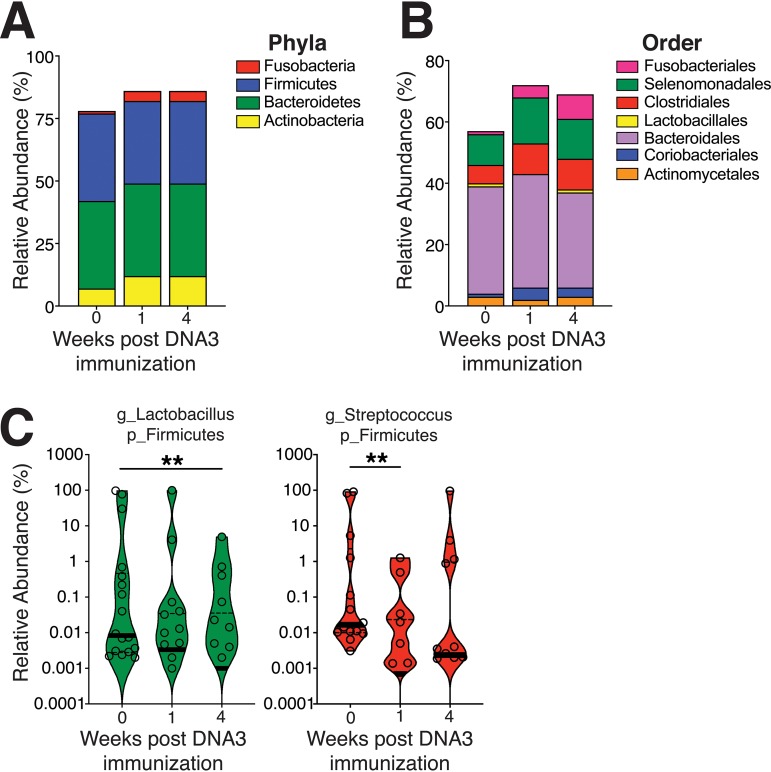
Temporal dynamics in vaginal microbiome composition after HIV-1 DNA immunization. (A) Relative abundances of phyla following DNA3 immunization. (B) Relative abundance of specific orders of genera following DNA3 immunization. (C) Violin plots show transient decrease in the genus *Lactobacillus* (g_*Lactobacillus*) and genus Streptococcus (g_*Streptococcus*) (adjusted *P* value of <0.05). The thick black line indicates the median, and the dashed lines show interquartile range. Significance was tested by Shannon alpha-diversity by group and time, adjusting for time since last menses and paired housing status. p_*Firmicutes*, phylum *Firmicutes*.

The composition of the vaginal microbiome in humans is influenced by a variety of factors, predominantly ethnicity, reproductive stage, status of menstrual cycle, pregnancy, and pelvic inflammatory disorders (30–33). However, the influence of vaccination on the microbiome of the vagina is yet to be determined. We explored the potential modulatory role of vaccination on the vaginal microbiome by first assessing whether any changes in microbial composition were evident at the phylum level and found the compartment to be relatively stable with a predominance of *Firmicutes* and *Bacteroidetes* phyla and the dominant orders (Fig. 3A and B).

At the genus level, 2 of the top 20 most abundant genera, *Lactobacillus* and *Streptococcus*, showed temporal changes following vaccination, with the relative abundance of *Lactobacillus* decreasing at week 4 post third DNA immunization and *Streptococcus* showing a transient decline at week 1 post third DNA immunization (Fig. 3C). A decrease in lactic acid producers could be a consequence of vaccine-induced inflammatory response. Indeed, SIV-infected alcohol-treated macaques displayed higher levels of white blood cells within the vaginal vault and showed a decrease in vaginal *Lactobacillus* (34, 35). We next constructed a multiple linear model, controlling for menstrual cycle, cohousing, and sibship to determine associations between bacterial genera in CVL and HIV-1 gp140 antibody responses. We found that the relative abundance of the Gram-positive, facultative anaerobe *Atopobium* (36) at week 4 after DNA3 immunization was negatively correlated with week 8 serum titers after the second protein boost (*r* = −0.47, *P* < 0.05; Fig. S5). Collectively, the data show that the microbiome of the vaginal tract was highly polymicrobial and remained largely stable over the course of immunization.

10.1128/mSphere.00824-19.5 FIG S4 Hierarchical clustering dendrogram using Bray distances by time since last menses at week 0 shows no significant association with microbiome composition. Darker pink indicates shorter times, and darker blue indicates longer times. Download FIG S4, TIF file, 0.7 MB. Copyright © 2019 Elizaldi et al. 2019 Elizaldi et al. This content is distributed under the terms of the Creative Commons Attribution 4.0 International license.

10.1128/mSphere.00824-19.6 FIG S5 Association between *g_Atopobium* and serum HIV-1 gp140 titers (*n* = 18; 2 animals with relative abundance *g_Atopobium* of 0 were excluded). Significance was tested by Spearman rank correlation test. Download FIG S5, TIF file, 0.9 MB. Copyright © 2019 Elizaldi et al. 2019 Elizaldi et al. This content is distributed under the terms of the Creative Commons Attribution 4.0 International license.

### Compositional plasticity in the rectal microbiome following HIV-1 DNA immunization.

**FIG 4:**
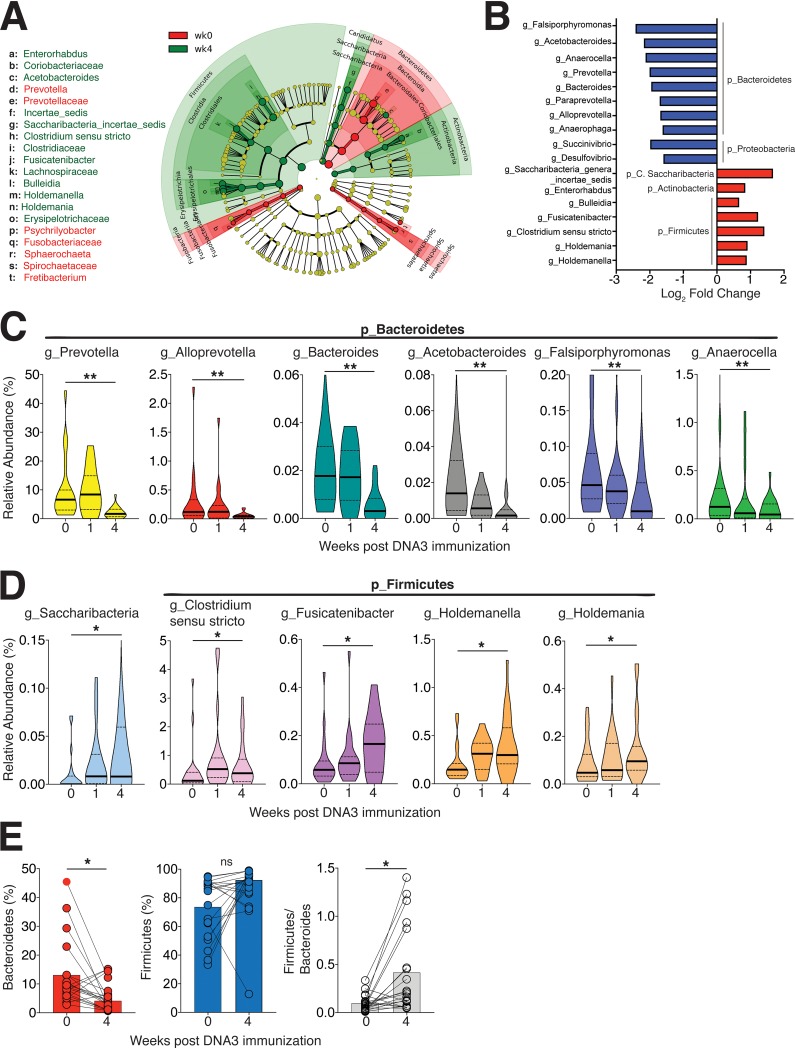
Compositional plasticity in the rectal microbiome after HIV-1 DNA immunization. (A) Cladogram representing taxa significantly altered at week 4 after DNA3 immunization relative to week 0 by LEfSe analysis. Statistical analysis of taxa was performed using Limma-Voom differential expression package in R and genera with an adjusted *P* value of <0.05 were included in LEfSe analysis. (B) Top 10 downregulated (blue) and upregulated (red) genera at week 4 relative to week 0. (C and D) Violin plots show genera significantly decreased (C) and genera significantly increased (D) following DNA3 immunization. The thick black line indicates median, and the dashed lines show interquartile range. (E) Decrease in *Bacteroidetes* and increase in *Firmicutes/Bacteroidetes* ratio at week 4 relative to week 0 (*P* < 0.05, tested using the Mann-Whitney U test). Significance was tested by Spearman rank correlation test. ns, not significant.

Having observed temporal stability in the vaginal microbiome following DNA3 immunization, we next sought to understand the effects of HIV-1 DNA immunization on the rectal microbiome. We performed multiple linear regression analysis using Limma-Voom in R adjusting for repeated measures and potential covariates as described in the methods section. This approach involved a false-discovery rate estimation to correct for multiple-hypothesis testing (37) and revealed dynamic changes in a total of 133 taxa (adjusted *P* value of <0.05; Table S1) at week 4 relative to week 0 after DNA3 immunization. From this list, we selected taxa with log_2_ fold change values above 0.5 and below −0.5 to generate a LEfSe plot (Fig. 4A). As depicted in Fig. 4B, seven taxa, the majority within the *Firmicutes* phyla, showed a relative increase in abundance (log_2_ fold change, 0.6 to 1.6), while the majority of genera less abundant at week 4 relative to week 0 fell within the *Bacteroidetes* phylum.

10.1128/mSphere.00824-19.1 TABLE S1 List of genera in rectal microbiome significantly different at week 4 relative to week 0. Download Table S1, XLS file, 0.04 MB. Copyright © 2019 Elizaldi et al. 2019 Elizaldi et al. This content is distributed under the terms of the Creative Commons Attribution 4.0 International license.

Among the downregulated genera, we found that 126 displayed log_2_ fold changes between −0.5 to −2.4 with a phylum distribution as follows: 37% within *Firmicutes*, 29% within *Proteobacteria*, and 13% within *Bacteroidetes*. The class *Clostridia* within *Firmicutes*, *Gammaproteobacteria* within *Proteobacteria*, and *Bacteroidia* within *Bacteroidetes* were major constituents that decreased following immunization.

We identified that specific genera in the *Bacteroidetes* phylum were decreased following DNA3 immunization, including *Prevotella*, *Alloprevotella*, *Bacteroides*, *Acetobacteroides*, *Falsiporphyromonas*, and *Anaerocella* (Fig. 4C). In contrast, abundance of *Saccharibacteria*, *Clostridium sensu stricto*, *Fusicatenibacter*, *Holdemanella*, and *Holdemania* genera increased from 0 to 4 weeks after DNA3 immunization (Fig. 4D). These dynamic changes resulted in a significant decrease in the relative abundance of *Bacteroidetes* phylum and a relative increase in the *Firmicutes*/*Bacteroidetes* ratio at week 4 (Fig. 4E)—a microbiome signature associated with low-grade systemic inflammation such as that observed with obesity (26, 38). These data imply that HIV-1 DNA immunization can modulate rectal microbiome composition.

### Association between rectal microbiome composition and HIV-1 antibody responses.

We ascertained that serum antibody following protein boost did not significantly associate with antibody induced following the DNA3 prime (Fig. S6). Next, we investigated whether specific bacterial taxa within the rectal compartment associated with HIV-1-specific antibody at the effector and memory time points following the protein boost. Previous studies of humans have linked the abundance of gut lactic acid bacteria to vaccine-induced IgA in the rectal compartment (39); thus, we hypothesized that abundance of *Lactobacillus* would correlate with rectal HIV-1 IgA responses.

10.1128/mSphere.00824-19.7 FIG S6 Serum gp140 IgG responses after protein immunization are not associated with serum gp140 IgG titers induced after DNA3 immunization. Download FIG S6, TIF file, 1.5 MB. Copyright © 2019 Elizaldi et al. 2019 Elizaldi et al. This content is distributed under the terms of the Creative Commons Attribution 4.0 International license.

To test this hypothesis, we used multiple linear regression, adjusting for potential confounders and repeated measures, to identify genera associated with HIV-1 gp140 antibody responses at the corresponding effector (2 weeks after protein) and memory (8 to 16 weeks after protein) time points. These time points were selected *a priori*, as they represent antibody derived from the short-lived plasmablast and the long-lived germinal center response, respectively.

**FIG 5:**
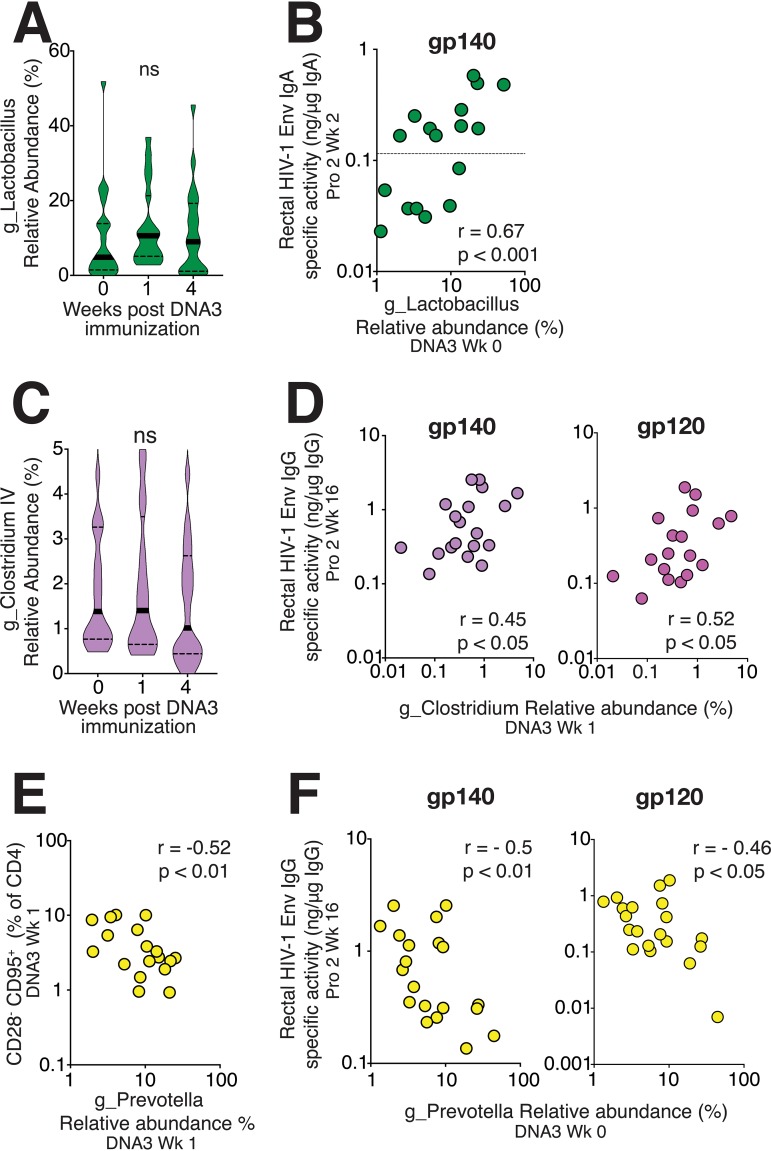
Association between rectal microbiome composition and HIV-1 antibody responses. (A) Violin plots show stability of rectal *Lactobacillus* over DNA immunization. (B) Relative abundance of *Lactobacillus* at DNA3 week 0 correlates with HIV-1 gp140 IgA in rectal secretions. (C) Violin plots show the relative abundance of *Clostridium* IV over time (D) and correlation between *Clostridium* IV and rectal gp140 IgG, and gp120 IgG levels. (E) Frequency of CD28^−^ CD95^+^ CD4 T cells at week 1 after DNA3 immunization correlate with contemporaneous abundance of *Prevotella.* (F) *Prevotella* abundance is a negative predictor of HIV-1 gp140 IgG and gp120 IgG in rectal secretions. Significance was tested by Spearman rank correlation test.

While relative rectal *Lactobacillus* abundance was not altered over the course of immunization (Fig. 5A), we found that *Lactobacillus* exhibited significant positive correlations with HIV-1-specific Env IgA at 2 weeks after the second protein boost (*r* = 0.69, *P* < 0.001; Fig. 5B). *Lactobacillus* abundance was also correlated with HIV-1 gp140 IgA induced following the first protein boost (data not shown; *r* = 0.4, *P* < 0.05). Moreover, studies of rhesus macaques associate *Lactobacillus* supplementation with a higher proportion of colonic IL-23+ antigen-presenting cells, which could underlie the observed associations (20). The lack of association of *Lactobacillus* with total IgA in rectal secretions could be explained by specificity to HIV-1-elicited mucosal IgA (Fig. S7A).

Similar to *Lactobacillus*, relative abundance of *Clostridium* IV remained unchanged after DNA immunization (Fig. 5C). We identified abundance of *Clostridium* IV (*r* = 0.45, *P* < 0.05) as a significant positive correlate of rectal HIV-1-specific gp140 and gp120 IgG concentrations at week 16; *Clostridium* IV did not correlate with total IgG in rectal secretions (Fig. 5D and Fig. S7B). This association is noteworthy considering that *Clostridium* spp. within clusters XIV and IV are reported strong inducers of Treg differentiation and accumulation (10) and promote expression of IL-10 in Tregs in colonic epithelial cells (10, 40). Emerging evidence for Tregs in supporting plasma cell differentiation could explain the observed associations (41).

10.1128/mSphere.00824-19.8 FIG S7 (A) Relative abundance of *g_Lactobacillus* at DNA3 week 0 does not correlate with total IgA in rectal secretions (B) Relative abundance of g_*Clostridium IV* does not correlate with rectal total IgG concentrations. Download FIG S7, TIF file, 1.2 MB. Copyright © 2019 Elizaldi et al. 2019 Elizaldi et al. This content is distributed under the terms of the Creative Commons Attribution 4.0 International license.

Because the relative abundance of *Prevotella* decreased following immunization, we sought to understand whether this decrease correlated with a specific gut-homing CD4 or monocyte subset. Our analysis revealed that frequencies of effector memory CD4 T cells were negatively associated with *Prevotella* abundance (*r* = −0.52, *P* < 0.01; Fig. 5E) as was the α_4_β_7_^+^ effector CD4 subset (data not shown; *r* = −0.4, *P* < 0.05). Unlike *Clostridium* IV, the abundance of *Prevotella* at week 0 correlated negatively with gp140- and gp120-specific HIV-1 IgG in rectal secretions (Fig. 5F).

Because intestinal microbiota can elicit antibodies that cross-react with the gp41 ectodomain of HIV envelope (42), we ascertained whether these specific microbiota correlated with baseline (i.e., preimmunization) variation in gp140 antibody concentrations within rectal secretions. The lack of significant associations between *Lactobacillus*, *Clostridium* IV, and *Prevotella* and preimmunization concentrations of gp140 IgG in rectal secretions suggested specificity of this association to vaccine-elicited antibody levels in the rectal mucosa (Fig. S8A to C).

10.1128/mSphere.00824-19.9 FIG S8 (A to C) Baseline gp140 antibody levels in rectal secretions are not associated with rectal microbiota. (D and E) Association between serum gp120 IgG antibody levels and specific rectal microbiota. Download FIG S8, TIF file, 2.6 MB. Copyright © 2019 Elizaldi et al. 2019 Elizaldi et al. This content is distributed under the terms of the Creative Commons Attribution 4.0 International license.

Additionally, we determined whether serum antibody responses to the gp120 protein at peak and memory time points associated with abundance of *Lactobacillus*, *Clostridium* IV, and *Prevotella*. The data revealed that serum IgG responses elicited against monomeric gp120 at week 8 after the second protein boost positively associated with *Clostridium* IV and negatively associated with *Prevotella* (Fig. S8D and E). No association between rectal microbiota and vaginal gp140 antibody levels were observed. Collectively, our data show that dynamic changes within the rectal microbiome occur following DNA3 immunization and that specific taxa within the rectal compartment associate with humoral immune responses after DNA immunization.

**FIG 6:**
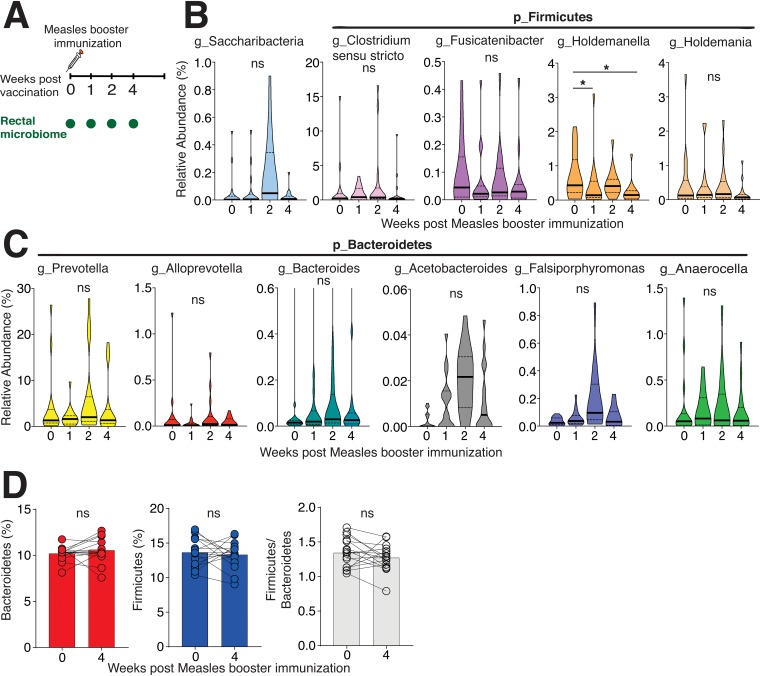
Rectal microbiome composition after measles booster vaccination. (A) Study overview. Female adult rhesus macaques (*n* = 16) were boosted with measles vaccine (MV), and rectal sponges were collected at weeks 0, 1, 2, and 4 relative to MV immunization. (B) Violin plots shows genera upregulated after HIV-1 DNA immunization being stable following MV immunization with the exception of *Holdemanella* which was significantly decreased at week 1 and week 4 following MV (adjusted *P* value of <0.05). (C) Violin plots show genera downregulated following HIV-1 DNA immunization were stable following MV immunization. (D) Stability of *Bacteroidetes* and *Firmicutes*. No change in the *Firmicutes*/*Bacteroidetes* ratio following measles immunization was observed. Significance was tested by Shannon alpha-diversity by group and time, adjusting for time since last menses and paired status.

On the basis of these observations, we next asked whether similar changes to the rectal microbiome composition would be observed following a booster measles vaccine in adult female monkeys (Fig. 6A). The rectal microbiome data from this second cohort of 16 animals had 346 OTUs and a read range of 10,744 to 111,051. *Firmicutes* was the most dominant phylum, similar to the first cohort (Fig. S9). However, in contrast to microbiome dynamics following HIV-1 DNA immunization, we found that the rectal microbiome remained largely stable with no significant changes in the majority of taxa across the rectal sampling period. We did observe that relative abundance of the genera *Holdemanella* (phylum *Firmicutes*) was significantly decreased at week 1 (log_2_ fold change of −1.67, adjusted *P* of <0.05) and week 4 (log_2_ fold change of −1.8, adjusted *P* of <0.05; Fig. 6B). The trajectory of *Holdemanella* was opposite that observed with HIV-1 DNA vaccination, indicating that dynamic changes in specific taxa and the resulting increased *Firmicutes/Bacteroidetes* ratio was specific to the HIV-1 immunization platform (Fig. 6C and D). These findings indicate that measles booster immunization does not induce significant perturbations to the rectal microbiome.

10.1128/mSphere.00824-19.10 FIG S9 (A) Rectal phyla abundance of 16 animals in measles study. (B) Body weight over the course of microbiome sampling following measles booster immunization. No differences in body weight were observed using a mixed-effects analysis of variance (ANOVA) model. (C) Robust boost in antibody responses following measles booster immunization (***, *P* < 0.0001 with a mixed-model ANOVA test at week 2 and week 4 relative to week 0). (D) Hierarchical clustering dendrogram using Bray distances by body weight for rectal microbiome at week 0. Darker pink indicates lower body weight, and blue indicates higher body weight. (E) Body weight over the course of microbiome sampling following DNA3 immunization. No differences in body weight were observed using a mixed-effects ANOVA model. Download FIG S9, TIF file, 1.7 MB. Copyright © 2019 Elizaldi et al. 2019 Elizaldi et al. This content is distributed under the terms of the Creative Commons Attribution 4.0 International license.

**FIG 7:**
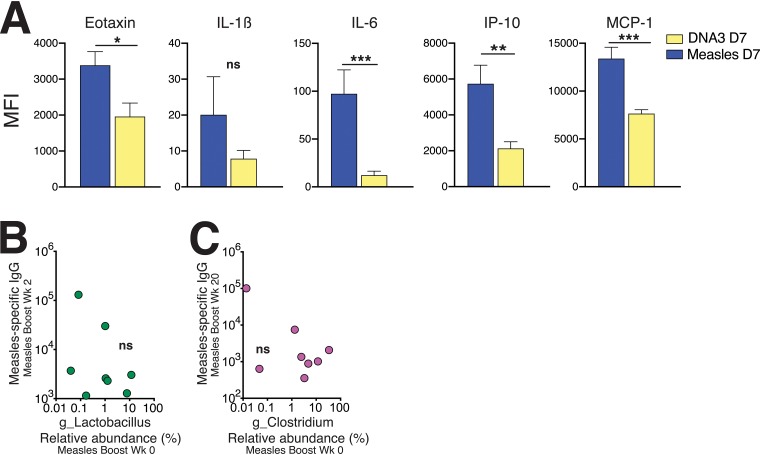
Serum cytokine profiles after DNA versus measles immunization. (A) Comparison of cytokine levels in serum on day 7 (D7) after DNA3 immunization and day 7 after measles immunization showed significantly higher levels of eotaxin, interleukin-1β (IL-1β), interleukin-6 (IL-6), interferon protein 10 (IP-10), and monocyte chemotactic protein 1 (MCP-1) following DNA3 immunization. Significance was determined using a Mann-Whitney test. MFI, mean fluorescent intensity. (B) Relative abundance of *Lactobacillus* was not associated with measles virus-specific antibody titers at week 2. (C) Relative abundance of *Clostridium* IV was not associated with measles virus-specific antibody titers at week 20.

Next, we determined whether the curtailed inflammatory milieu following measles immunization might explain the lack of observed changes in rectal microbiome composition. To this end, we compared cytokine levels across HIV-1 DNA and measles vaccine regimens (Fig. 7A). The data showed that levels of proinflammatory cytokines were significantly lower at day 7 following measles immunization relative to the DNA3 immunization, suggesting a possible link between inflammation and changes to the rectal microbiome following the third DNA immunization. Furthermore, the abundance of *Lactobacillus* and *Clostridium* IV did not correlate with measles-specific IgG responses in sera, indicating specificity of these associations to HIV-1 Env antibody responses (Fig. 7B and C).

In summary, we find that the relative proportion of *Bacteroidetes* is decreased following DNA3 immunization, resulting in an increase in the *Firmicutes*/*Bacteroidetes* ratio. Together with the associations between specific genera and antibody responses, our findings indicate that immune responses following HIV-1 DNA immunization induce perturbations within the rectal microbial compartment, which could have implications for vaccine design.

## DISCUSSION

This study allows for three main conclusions. First, the vaginal microbiome and rectal microbiome are colonized by distinct microbial taxa during homeostasis and respond differentially to an HIV-1 DNA immunization. While the vaginal microbiome remained relatively stable, several genera in the *Bacteroidetes* and *Firmicutes* phyla were altered within the rectal microbiome, resulting in an increased *Firmicutes*/*Bacteroidetes* ratio. Second, the relative abundance of *Lactobacillus* and *Clostridium* IV in the rectal compartment were strong positive correlates of local HIV-1-specific IgA and IgG levels, respectively. Third, a decrease in *Prevotella* species was associated with increased frequencies of gut-homing CD4 T cells after vaccination and *Prevotella* abundance was a negative correlate of rectal HIV-1 IgG antibody. Collectively, our observations provide a rationale to target the mucosal microbiome as a strategy to enhance vaccine-induced immune responses and, ultimately, vaccine efficacy.

Microbiome community structure is determined by a variety of factors depending on the anatomical location. In adult primates, diet is a major driver of gut microbiome structure (27, 43, 44), while compositional changes in the vaginal microbiome occur with pregnancy, menstruation, and inflammatory conditions, such as bacterial vaginosis (45–47). Consistent with observations in cynomolgus macaques, we found that in rhesus macaques, vaginal and rectal microbiome composition was clearly distinct at the genus level while sharing phylum-level similarities (48). Bacterial phylum-level concordance across these distinct mucosal compartments is linked to vertical bacterial transmission during birth (27), although the specifics for colonization of the various microbial communities remain to be determined.

There is evidence to suggest that the rectal microbiome may be more amenable to external influences. For example, measurement of vaginal and rectal *Lactobacillus* spp. in 31 healthy pregnant women showed a dramatic decrease of *Lactobacillus* within the rectal compartment after the first trimester, while vaginal *Lactobacillus* spp. were relatively stable (49). The stability of the vaginal microbiome following immunization may reflect a lack of significant immune activation at the vaginal portal following the third DNA immunization. However, the marked taxonomical variability within the vaginal microbiota could also mean that larger sample sizes are needed to capture differences over time. These fundamental questions remain open, and further studies are needed to rigorously address whether vaccine-specific cells infiltrate the female genital tract and whether more immunogenic vaccine modalities have the capacity to influence vaginal microbial composition.

An important observation from our study was that relative abundance of *Lactobacillus* and *Clostridium* IV positively correlated with rectal HIV-1 IgA and IgG antibody levels. There is compelling evidence in humans and nonhuman primates that the microbiome impacts humoral immune responses to vaccination (17, 18). The abundance of lactic acid-producing bacteria, such as *Bifidobacteria*, positively correlates with cellular and antibody responses to oral rotavirus vaccination in infants (50). On the other hand, increased relative abundance of *Enterobacteriales*, *Pseudomonadales*, and *Clostridiales* relative to *Bifidobacteria* is associated with lower vaccine-specific responses, suggesting that higher relative abundance of *Bifidobacteria* might promote vaccine responses. Similarly, abundance of fecal *Actinobacteria* (*Bifidobacterium* spp.) was associated with higher poliovirus- and tetanus toxoid-specific T cell responses and higher poliovirus-specific IgG levels in children’s serum (51). These studies collectively support the hypothesis that certain bacterial species within *Actinobacteria* and *Firmicutes* phyla augment immune responses to vaccination, while genera within the *Bacteroidetes* phylum may impair host responses to vaccines. Furthermore, a randomized double-blind placebo control study in infants found that fermented infant formula, which favored intestinal *Bifidobacteria*, was associated with higher fecal poliovirus-specific IgA (52). Collectively, these data support the contention that the rectal microbiome composition can impact IgG and IgA, local and systemic, antibody responses to both vaccination and infection.

The mechanisms by which *Lactobacillus* spp. and other lactic acid-producing bacteria, such as *Bifidobacterium*, influence humoral immunity remain to be delineated. However, several possibilities central to lactic acid production can be explored. First, although lactic acid is largely considered a terminal metabolite of glycolysis, lactate can reenter the glycolytic pathway to generate acetyl coenzyme A (acetyl CoA) to support the Krebs cycle for energy generation, thereby fueling effector/plasmablast proliferation (53). Second, increased lactic acid can enhance memory cell differentiation. Lymphocytes express lactic acid transporters on their cell surface, and lactate-mediated inhibition of aerobic glycolysis could favor greater shunting of effector cells into the memory pool, which would subsequently favor greater recall B cell responses (54, 55). Third, lactate could have immunomodulatory effects; lactate is shown to skew CD4 T cell differentiation to the T_h_17 phenotype in an IL-23-dependent manner, which could promote mucosal B cell responses (56–59). Thus, the functions of lactate as a metabolite and as an intrinsic immunomodulator may underlie the observed associations. Of note, antibody responses are determined by multiple factors not limited to optimal priming of innate immune cells, magnitude of the extrafollicular response, and the germinal center response. Therefore, further studies are needed to delve into interactions between microbiota and relevant immune parameters to understand the mechanistic basis of the associations between microbiota and humoral immunity.

While our reported observations are novel, our study has several drawbacks. First, our study was not designed to capture microbiota shifts following the first two DNA immunizations. It is therefore possible that specific taxa, reaching a new stasis following the initial two DNA primes, were resilient to inflammatory responses ensuing from the third DNA prime. On the other hand, taxa dynamically modulated following DNA3 may have achieved a new equilibrium prior to the protein boost. An additional point of import is that our measles virus-vaccinated cohort, being highly seropositive prior to the boost, does not make for a rigorous control group. Therefore, the reported shifts in rectal microbial composition can be interpreted only relative to the third DNA immunization, and this significantly limits extrapolation of our results. Thus, more comprehensive analyses are needed to accurately capture the dynamics of microbial shifts to DNA vaccination.

Second, we performed the studies with female adult rhesus macaques, and whether the observed associations also hold true across the age spectrum and in males is an important consideration for future studies. Third, our animals were free-feeding and although they maintained stable body weight over the microbiome sampling period (see Fig. S9 in the supplemental material), we cannot entirely exclude the contribution of diet or other unmeasured confounding factors to the observed changes over time. Fourth, rectal immune cellular responses were not measured, and additional in-depth studies are needed to link mucosal microbiome with mucosal cellular immune responses. Another consideration is that the intestinal microbiota could have primed antibodies cross-reactive to the HIV-1 envelope (42), and the subsequent boosting of these antibodies by vaccination could underlie the observed associations. Therefore, further in-depth studies are needed to determine the basis of our observed associations between rectal microbiota and HIV-1 Env antibody concentrations.

In summary, we have identified specific microbial taxa in the rectal microbiome that associate with HIV-1 antibody responses. While the underlying mechanisms remain unclear, this is the first study to report an association between microbiome and humoral immunity to HIV-1. This study lays the foundation to design future mechanistic studies to delineate host intrinsic factors influencing HIV-1 vaccine efficacy. Our data also emphasize the need to stratify animals based on enterotypes or cervicotypes in macaque studies to obtain more robust measures of HIV vaccine immunogenicity and efficacy. To conclude, the significance of this study is in highlighting the impact of the mucosal microbiome on vaccine-induced immune responses. Considering that the rectal compartment is the most susceptible of the mucosal portals to HIV acquisition, our findings have significant implications for microbial manipulation as a strategy to improve HIV vaccine efficacy.

## MATERIALS AND METHODS

### Ethics statement.

This study was approved by the Institutional Animal Care and Use Committee at the University of California, Davis. All experiments were conducted in strict accordance with USDA regulations, and the recommendations for conducting experiments are in accord with the highest scientific, humane, and ethical principles as stated in the *Guide for the Care and Use of Laboratory Animals* (60).

### Rhesus macaques.

Adult female colony-bred Indian rhesus macaques (Macaca mulatta) housed at the California National Primate Research Center were used for both studies. The animals were negative for simian immunodeficiency virus (SIV), simian T lymphotropic virus (STLV), and simian retrovirus (SRV), and they did not have a history of pharmacological or dietary intervention with known influences on the microbiome. All animals were healthy, menstruating females with intact ovaries.

### Diet, housing, and antibiotics.

All animals received High-Protein Monkey Diet Jumbo (catalog no. 5047; LabDiet) with caloric distribution as follows: 30.3% protein, 13.2% fat, and 56.3% carbohydrate. The animals were housed in pairs in standard nonhuman primate cages. The animals received standard primate feed as described above as well as fresh fruit and enrichment daily and had free access to water. Immunizations, infections, blood draws, and biopsy procedures were performed under anesthesia by trained research staff. All efforts were made to schedule samples on paired animals concurrently in order to minimize potential distress. Animals in the study did not receive any antibiotics for at least 7 months prior to the sample collection for microbiome analysis.

### Study design and immunizations for HIV-1 DNA/protein vaccine study.

The study cohort consisted of adult females (*n* = 20) housed in stable pairs, indoors for at least 7 months prior to microbiome profiling. Animals were 4.29 ± 0.39 years (mean ± standard deviation [SD]) and had a mean body weight of 5.6 ± 0.92 kg at the initiation of microbiome sampling. We did not observe distinct clustering of week 0 rectal microbiome with body weight, and no significant changes in body weight were observed during the microbiome sampling period (Fig. S9E). DNA immunizations were delivered via intradermal injection with electroporation utilizing the ICHOR TriGrid Array (Ichor Medical Systems, San Diego, CA) at weeks 0, 8, and 16. For each DNA immunization, animals received 4 mg of the pGA2/JS2 plasmid DNA vector (61) encoding either simian-human immunodeficiency virus (SHIV) C.1086 T/F Env + interferon-induced protein 10 (IP-10) or SHIV C.1086 T/F Env alone. Protein immunization consisted of 100 μg C.ZA 1197MB gp140 protein (Immune Technology, New York, NY) adjuvanted with 100 μg monophosphoryl lipid A (MPLA) plus 50 μg QS-21 or with 100 μg MPLA plus 600 μg aluminum at weeks 30 and 44.

### Study design and immunizations for measles vaccine study.

The study cohort consisted of adult female rhesus macaques (*n* = 16) with a mean age of 12 ± 7.72 years. Animals were housed indoors for 3.5 to 100 months prior to microbiome profiling. Animals were immunized with the canine distemper virus-measles virus vaccine (Vanguard, Zoetis) at week 0 after collection of rectal sponges for microbiome analysis. The animals had a mean body weight of 7.1 ± 1.7 kg at the beginning of the study, and no significant changes in body weight were observed during the microbiome sampling period (Fig. S9B). All animals were seropositive for measles virus before booster immunization and showed anti-measles virus specific antibody responses after immunization, indicating a successful recall response (Fig. S9C).

### C.1086 gp140-specific IgG ELISA of serum and rectal secretions.

Serum IgG titers against HIV-1 C.1086 Env gp140 were determined by enzyme-linked immunosorbent assays (ELISAs) using standard protocols (21). Baseline sera from each animal served as negative control, and optical density (OD) values twofold above baseline were considered positive and extrapolated to determine anti-Env antibody concentrations. Antibody levels in rectal secretions were measured using a binding antibody multiplex assay using C.1086 gp140 K160N-labeled magnetic beads (MagPlex; Bio-Rad) as previously described (62). C.1086 gp120 (from B. Haynes) coated on the magnetic beads was used.

### Flow cytometry.

The distribution of T cell subsets in peripheral blood samples and their activation was determined by flow cytometry as previously described (23). T cell and monocyte subsets were characterized using the following antibodies: anti-CD3 (SP34-2), anti-CD4 (L200), anti-PD-1 (EH12.2H9), anti-ICOS (C398.4A), anti-CXCR3 (IC6), anti-CXCR5 (MU5UBEE), anti-CCR4 (1G1), anti-CCR6 (G034E3), anti-CD28 (CD28.2), anti-CD95 (DX2), anti-α_4_β_7_ (Act-1; NHP Reagent Resource), anti-OX40 (L106), anti-CD25 (BC96), anti-CD16 (3G8), anti-CD14 (M5E2), and anti-HLA-DR (L243). T cell phenotypes were defined by CD28 and CD95 markers: CD28^+^ CD95^−^ (naive), CD28^+^ CD95^+^ (central memory), and CD28^−^ CD95^+^ (effector memory). Monocyte subsets were defined as SSChi, HLA-DR^+^, CD14^+^/CD16^+^. All samples were collected on a BD FACSymphony flow cytometer with FACS Diva version 8.0.1 software. Compensation, gating, and analysis were performed using FlowJo v10.6.1. A Legendplex assay (Biolegend) was performed to evaluate cytokines in rhesus macaque sera. The assay was performed according to the manufacturer’s protocol. Samples were acquired on a BD LSR Fortessa cell analyzer.

### Microbiome profiling. (i) DNA extraction.

The rectal microbiota and vaginal microbiota were assessed in total DNA from rectal sponges and cervicovaginal lavage (CVL) samples, respectively. Rectal sponges were placed in 15-ml conical tubes and submerged with 1 ml of 1× phosphate-buffered saline (PBS) solution. The samples were vortexed thoroughly for 5 s and then briefly spun to collect contents at the bottom of the tube. One half of the volume (500 μl) was used for DNA extractions. Sample tubes containing CVL samples were thawed on ice, and one half of the volume in each tube (approximately 250 to 280 μl) was used for DNA extraction. DNA was isolated using the Qiagen DNeasy PowerSoil kit (Qiagen) with the following modifications. After the addition of buffer C1, samples were incubated at 65°C for 10 min and then subjected to homogenization using a Qiagen TissueLyser II (Qiagen) for 10 min at 20 cycles per second. The samples were turned 180 degrees and subjected to further homogenization for an additional 10 min at 20 cycles per second, per the manufacturer’s recommendation. Buffers C2 and C3 were combined at half the normal volume and incubated at 4°C for 10 min in a single step, rather than adding them individually in sequential steps. Samples were eluted in 60 μl of buffer C6.

**(ii) PCR amplification.** Amplification of the V3-V4 domain of the 16S rRNA gene was performed using a DNA template and primers 319F (F stands for forward) [TCGTCGGCAGCGTCAGATGTGTATAAGAGACAG(spacer)GTACTCCTACGGGAGGCAGCAGT] and 806R (R stands for reverse) [GTCTCGTGGGCTCGGAGATGTGTATAAGAGACAG(spacer)CCGGACTACNVGGGTWTCTAAT] using a two-step PCR procedure. In step one of the amplification procedure, both forward and reverse primers contained an Illumina tag sequence, a variable length spacer to increase diversity and improve the quality of the sequencing run, a linker sequence, and the 16S target sequence. Each PCR contained 1 U Kapa2G Robust Hot Start polymerase (Kapa Biosystems), 1.5 mM MgCl_2_, 0.2 mM final concentration deoxynucleoside triphosphate (dNTP) mix, 0.2 μM final concentration of each primer, and 1 μl of DNA for each sample. PCR conditions were as follows: an initial incubation at 95°C for 45 s, 50°C for 30 s, 72°C for 30 s, and a final extension of 72°C for 3 min. In step two, each sample was barcoded with a unique forward and reverse barcode combination using forward primers with an Illumina P5 adapter sequence, a unique 8-nucleotide (nt) barcode, a partial matching sequence of the forward adapter used in step one and reverse primers with an Illumina P7 adapter, unique 8-nt barcode, and a partial matching sequence of the reverse adapter used in step one. The PCR in step two contained 1 U Kapa2G Robust Hot Start polymerase (Kapa Biosystems), 1.5 mM MgCl_2_, 0.2 mM final concentration dNTP mix, 0.2 μM final concentration of each uniquely barcoded primer, and 1 μl of the product from the PCR in step one diluted at a 10:1 ratio in water. PCR conditions were as follows: (i) an initial incubation at 95°C for 3 min; (ii) 8 cycles, with 1 cycle consisting of 95°C for 30 s, 58°C for 30 s, and 72°C for 30 s; and (iii) a final extension step of 72°C for 3 min.

The final product was quantified on the Qubit instrument using the Qubit Broad Range DNA kit (Invitrogen) and individual amplicons were pooled in equal concentrations. The pooled library was cleaned utilizing Ampure XP beads (Beckman Coulter) then the band of interest was further subjected to isolation via gel electrophoresis on a 1.5% Blue Pippen HT gel (Sage Science). The library was quantified via qPCR followed by 300-bp paired-end sequencing using an Illumina MiSeq instrument in the Genome DNA Technologies Core, University of California, Davis.

### Bioinformatics.

All samples were sequenced on an Illumina MiSeq platform at the Genome Biosciences Facility at the University of California, Davis. Analysis began with demultiplexing sequence reads. Demultiplexing of the raw FASTQ files and adapter trimming of sequences were performed using dbcAmplicons version 0.8.5. (https://github.com/msettles/dbcAmplicons). The unmerged forward and reverse reads were imported into QIIME2 version 2017.12 (https://qiime2.org), and sequence variants were determined following the DADA2 analysis pipeline. Each sequence was assigned to its given samples based on the given barcode. Reads that did not match any barcode were discarded (failed to meet minimum quality thresholds). Barcoded forward and reverse sequencing reads were quality filtered and merged. Sequences that were only observed one time or only in a single sample were also discarded. Chimeras were detected and filtered from paired-end reads. After quality filtering, sequences are clustered into operational taxonomic units (OTUs). Comparison of clustered sequences were performed against a SILVA 132 reference database. All data generated in this study utilized the same instrumentation, technician reference database, packages, and pipeline.

### Data processing and filtering and trimming of reads.

The data were filtered as follows: ambiguous phyla were removed, phyla with a mean prevalence of less than 5 were removed, taxa were agglomerated at the genus level, all taxa without genus-level taxonomic assignments were removed, and samples with fewer than 10,000 reads were removed.

### Statistical analysis.

Statistical analysis of microbial community data was performed primarily using the Bioconductor package phyloseq (version 1.22.3, in R 3.4.4) (63). Hierarchical clustering dendrograms were calculated based on Bray-Curtis distances. Differential abundance analyses were performed using the limma-voom Bioconductor pipeline (limma version 3.34.9, edgeR version 3.20.9), following relative log expression (RLE) normalization (64, 65).

The models fitted were as follows: study 1 was HIV-1 DNA vaccine study, and study 2 was the measles vaccine study.

In study 1 CVL, the model included factors for time, treatment group, the time-treatment group interaction, cohoused pair, and time since last menses.

In study 1 rectal, the model included factors for time, treatment group, the time-treatment group interaction, and cohoused pair.

In study 2 rectal, the model included a factor for time.

Standard errors of log fold changes were adjusted for within-animal correlations. An adjusted *P* value of 0.05 was used to indicate statistical significance.

Shannon alpha-diversity was analyzed using linear mixed-effects models, with fixed effects as specified above for limma-voom and a random intercept for animal. Linear mixed-effects models were fitted using the R package nlme, version 3.1-137.

QIIME2 was used to calculate alpha-diversity metrics, such as observed OTUs, Shannon evenness, and beta-diversity, and weighted/unweighted UniFrac distances.

### Data availability.

All relevant data have been included in the article. We will provide any additional data upon request. Raw sequence data are available in the BioProject database under accession number PRJNA593065.
