# Supplementary figures and images for: Rectal Microbiome Composition Correlates with Humoral Immunity to HIV-1 in Vaccinated Rhesus Macaques
Source: mSphere. 2019 Dec 11;4(6):e00824-19. doi: 10.1128/mSphere.00824-19 (PMC6908426; doi:10.1128/mSphere.00824-19)

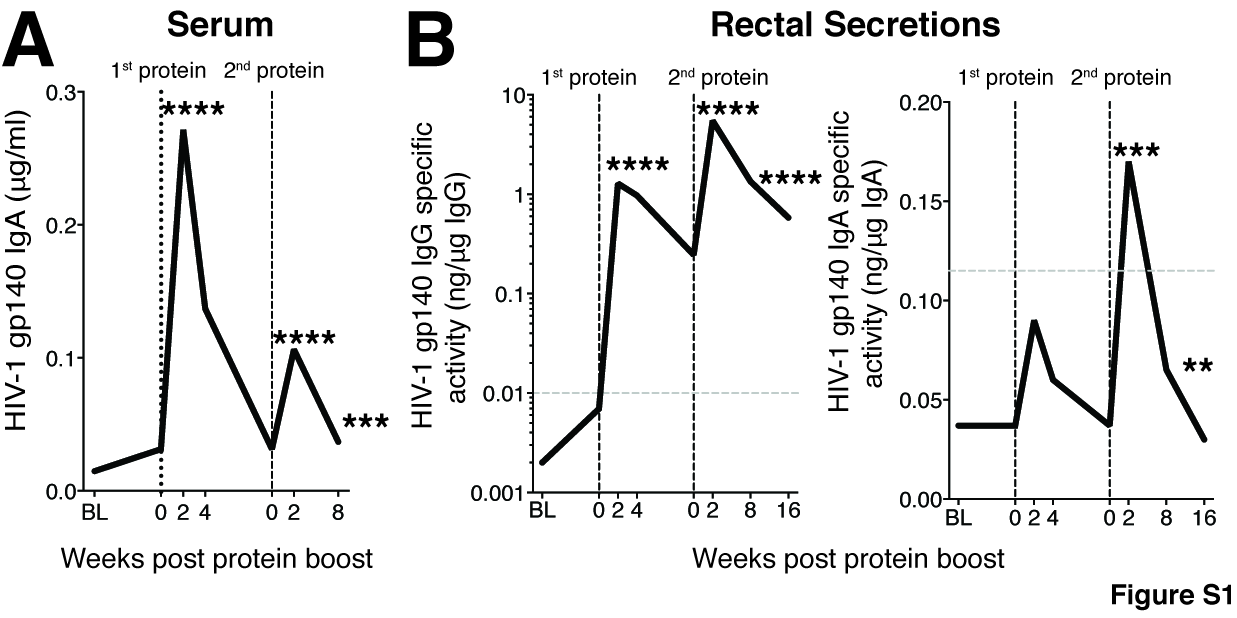

Supplement: FIG S1 [file mSphere.00824-19-sf001.tif]

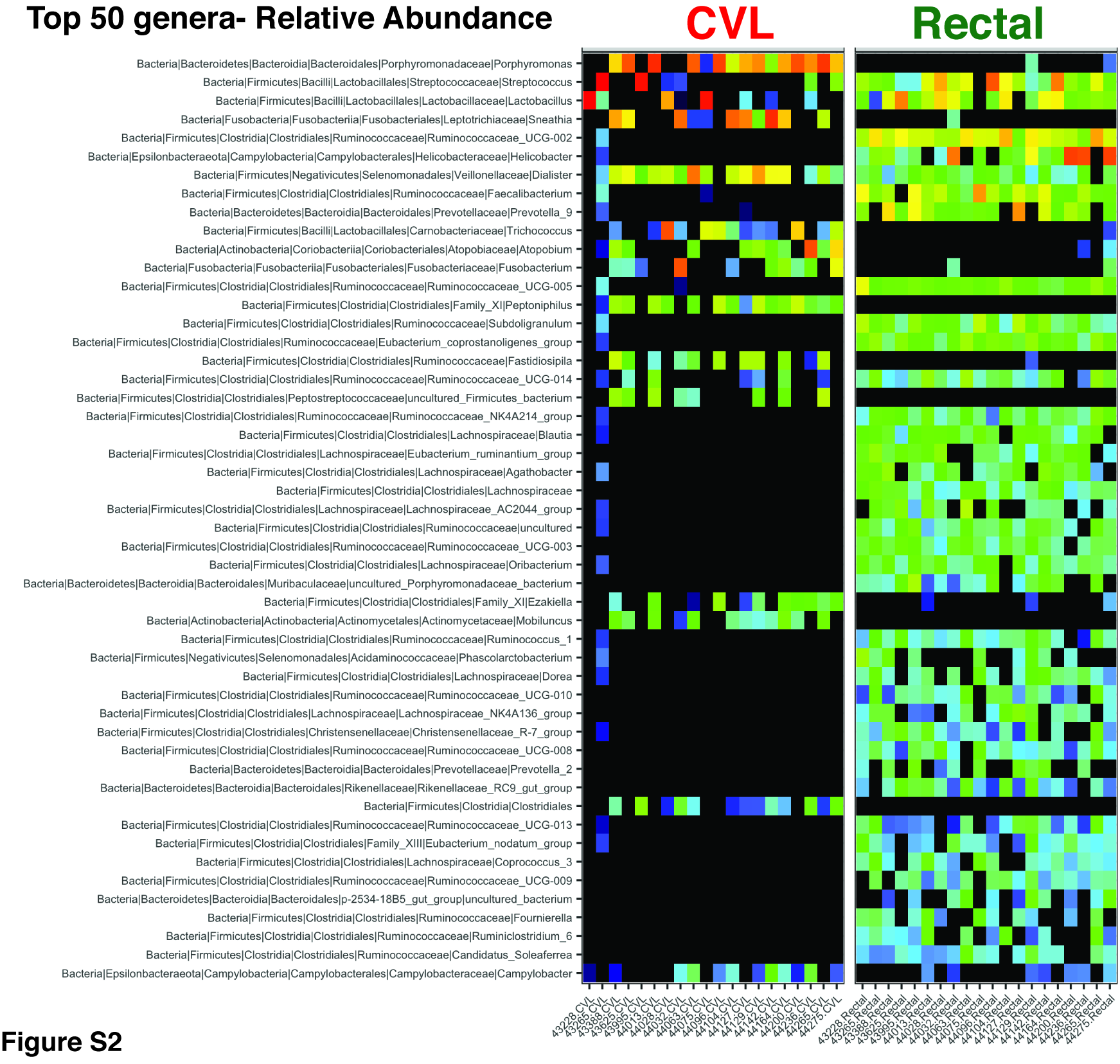

Supplement: FIG S2 [file mSphere.00824-19-sf002.tif]

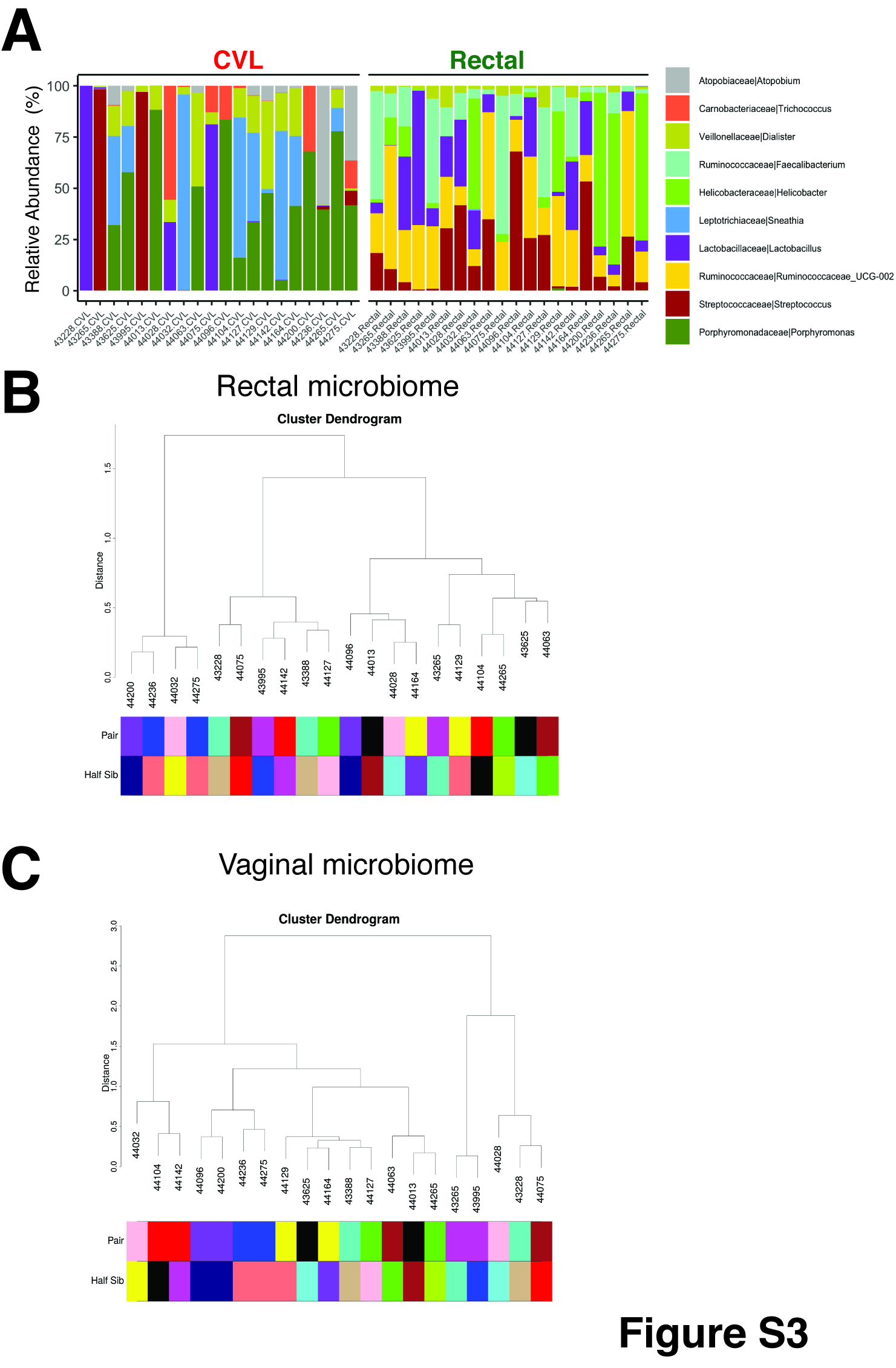

Supplement: FIG S3 [file mSphere.00824-19-sf003.tif]

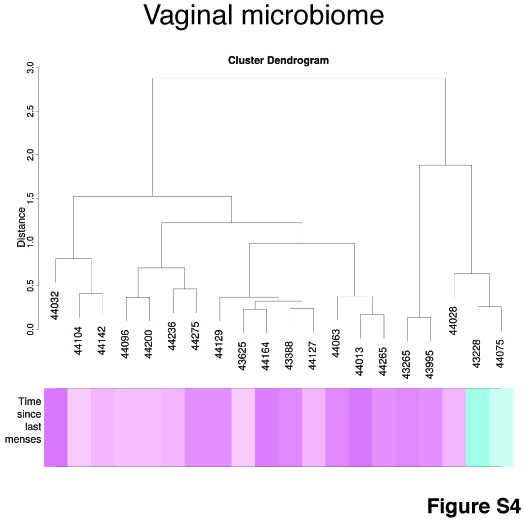

Supplement: FIG S4 [file mSphere.00824-19-sf004.tif]

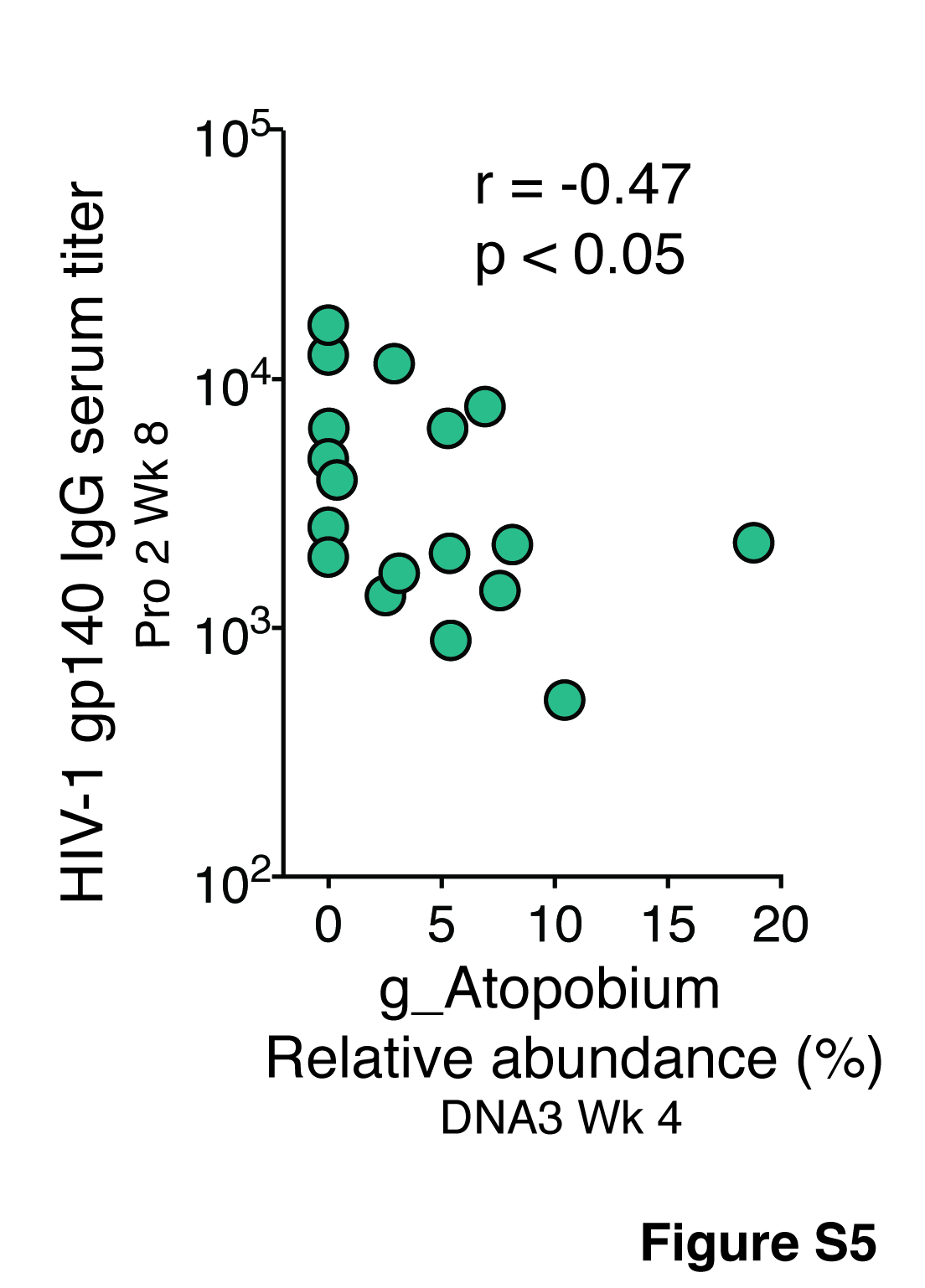

Supplement: FIG S5 [file mSphere.00824-19-sf005.tif]

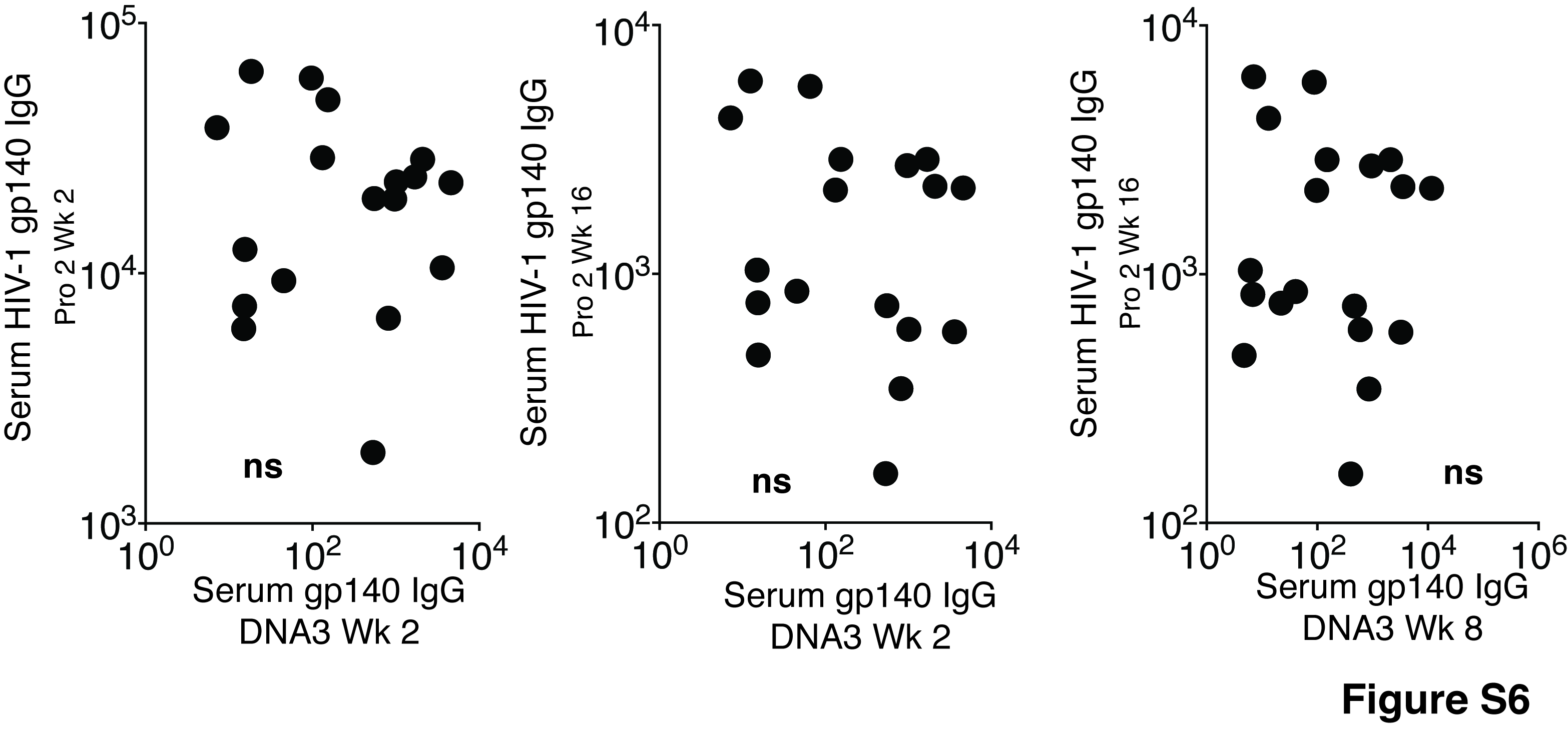

Supplement: FIG S6 [file mSphere.00824-19-sf006.tif]

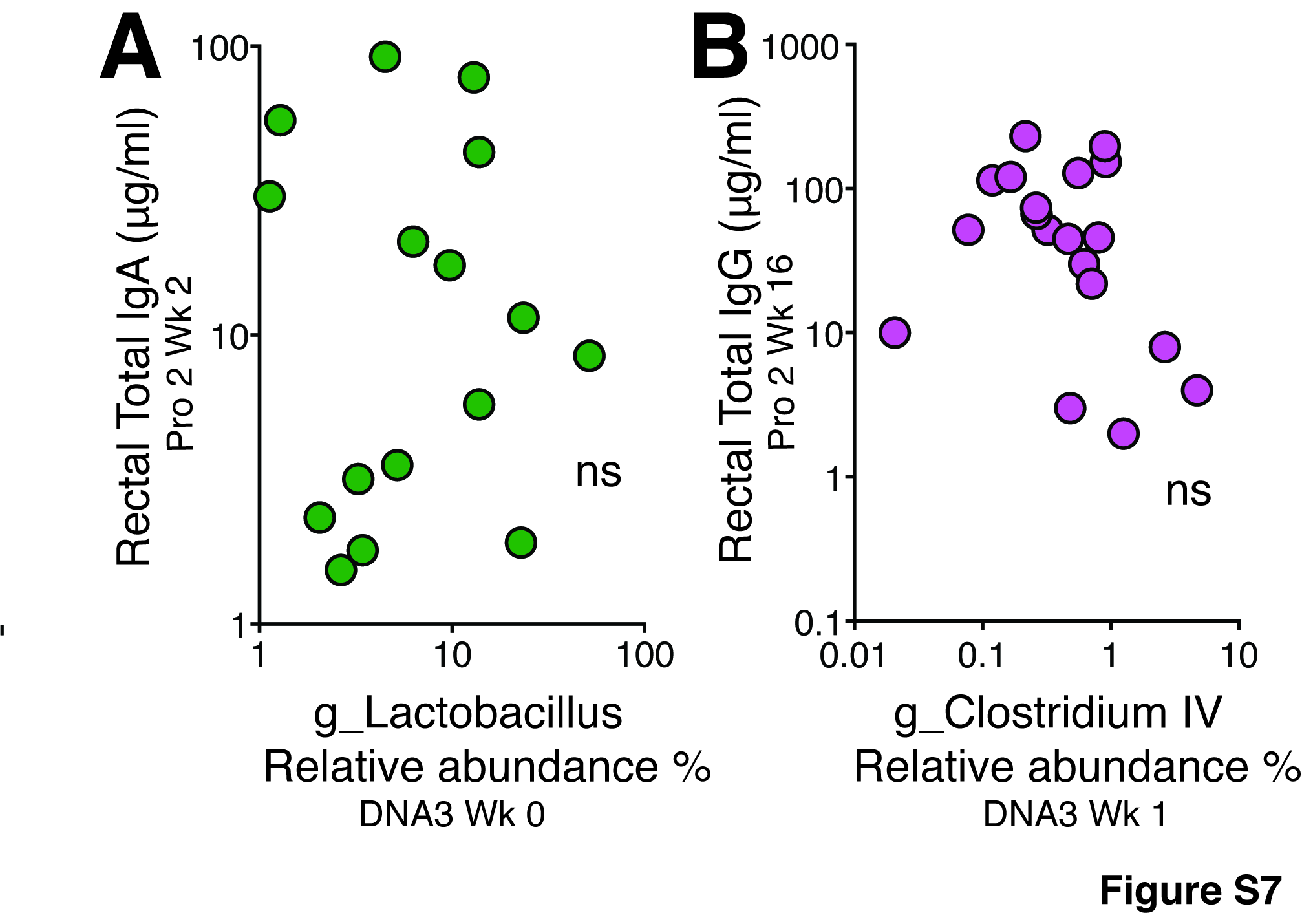

Supplement: FIG S7 [file mSphere.00824-19-sf007.tif]

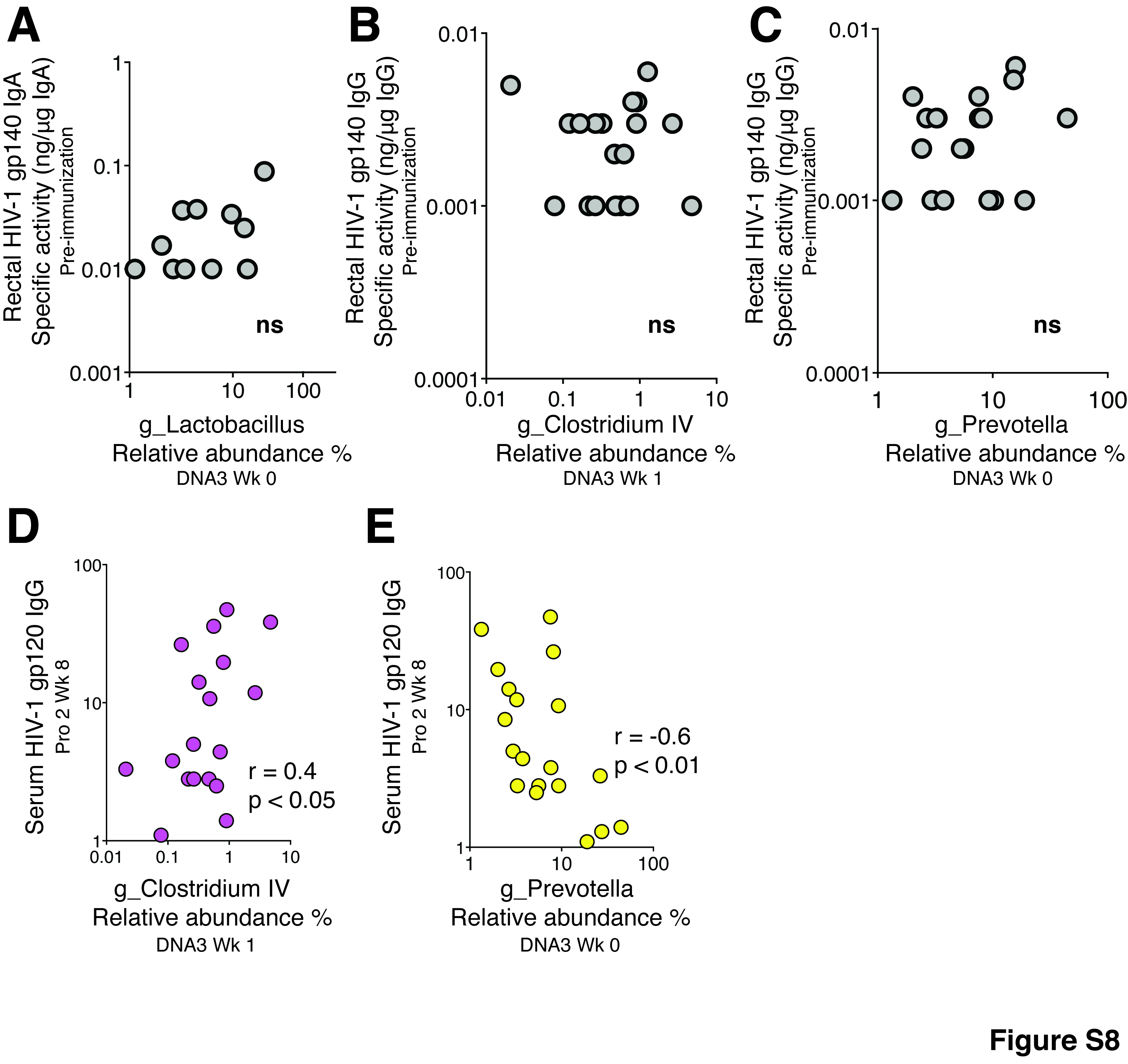

Supplement: FIG S8 [file mSphere.00824-19-sf008.tif]

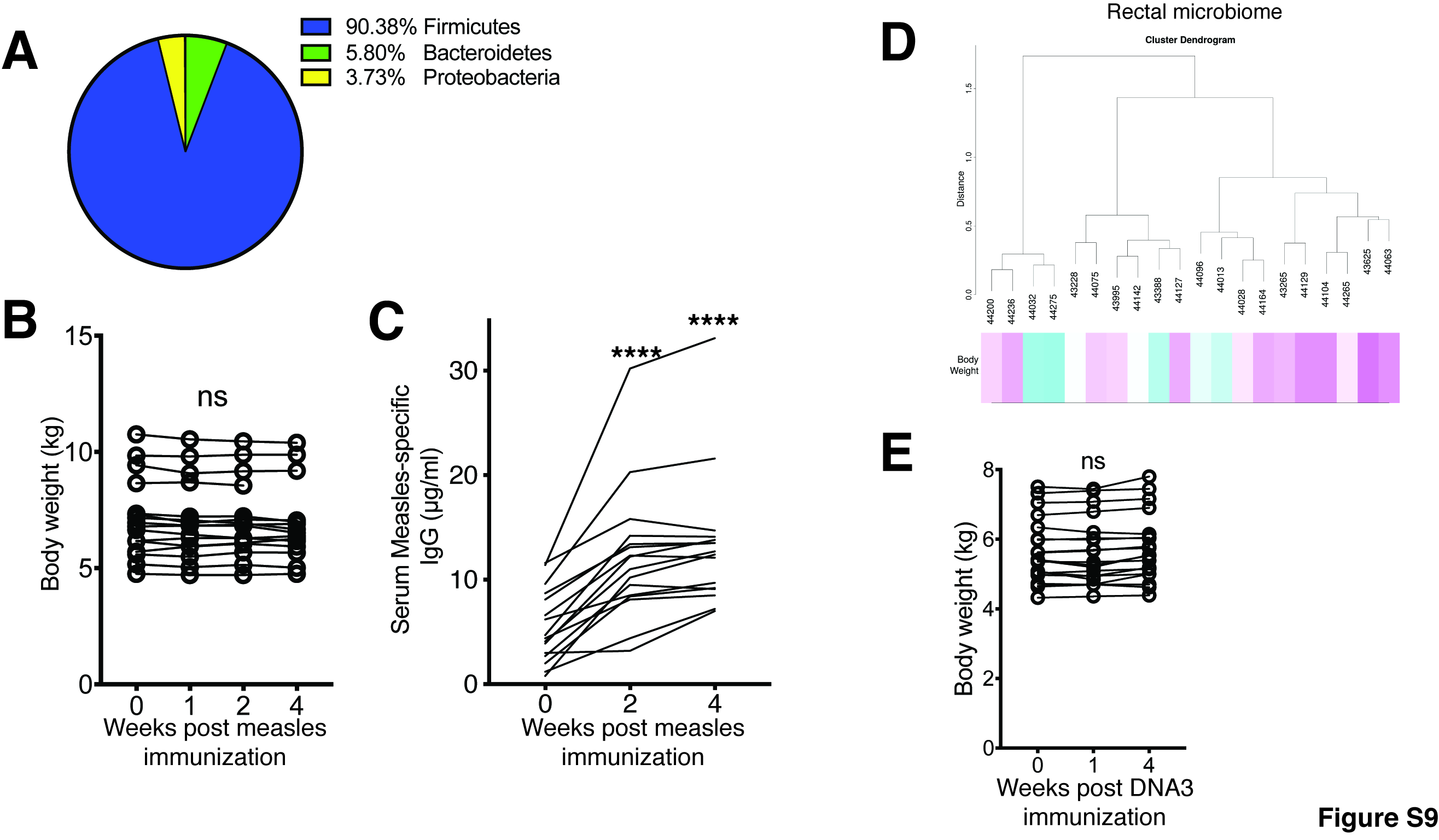

Supplement: FIG S9 [file mSphere.00824-19-sf009.tif]
